# Bis(η^5^-cyclo­penta­dien­yl)(2-{[(2-meth­oxy­phen­yl)imino]­meth­yl}phenolato-κ^3^ *O*,*N*,*O*′)terbium

**DOI:** 10.1107/S2056989021013025

**Published:** 2022-01-01

**Authors:** Mikhail E. Minyaev, Konstantin A. Lyssenko, Dmitrii M. Roitershtein, Ilya E. Nifant’ev

**Affiliations:** a N.D. Zelinsky Institute of Organic Chemistry, Russian Academy of Sciences, 47 Leninsky Prospect, Moscow, 119991, Russian Federation; b A.V. Topchiev Institute of Petrochemical Synthesis, Russian Academy of Sciences, 29 Leninsky prospect, Moscow, 119991, Russian Federation; c Chemistry Department, M.V. Lomonosov Moscow State University, 1 Leninskie Gory, Str., Building 3, Moscow, 119991, Russian Federation

**Keywords:** lanthanide, terbium, bis­(cyclo­penta­dien­yl), (meth­oxy­phenyl­imino­meth­yl)phenolate, salicyl­imino, crystal structure

## Abstract

The air- and moisture-sensitive title compound, [Tb(C_5_H_5_)_2_(C_14_H_12_NO_2_)], was synthesized from tris­(cyclo­penta­dien­yl)(tetra­hydro­furan)­terbium and 2-{[(2-meth­oxy­phen­yl)imino]­meth­yl}phenol. Each Tb atom is coordinated by two cyclo­penta­dienyl ligands in an η^5^-coordination mode and by one N and two O atoms of the organic ligand in a tridentate κ^3^ *O*,*N*,*O*′-mode.

## Abstract 2

The air- and moisture-sensitive title compound was synthesized from tris­(cyclo­penta­dien­yl)(tetra­hydro­furan)­terbium and 2-{[(2-meth­oxy­phen­yl)imino]­meth­yl}phenol. Each Tb atom is coordinated by two cyclo­penta­dienyl ligands in an η^5^-coordination mode and by one N and two O atoms of the organic ligand in a tridentate κ^3^ *O*,*N*,*O*′-mode.

## Chemical context

Bis(cyclo­penta­dien­yl) complexes of rare-earth metals attract significant attention because of their important role in the development of organometallic chemistry of 4*f* elements (Schumann *et al.*, 1995 ▸; Wedal & Evans, 2021 ▸; Evans, 2016 ▸). This type of complex is one of the first discovered organolanthanide classes (Maginn *et al.*, 1963 ▸). 
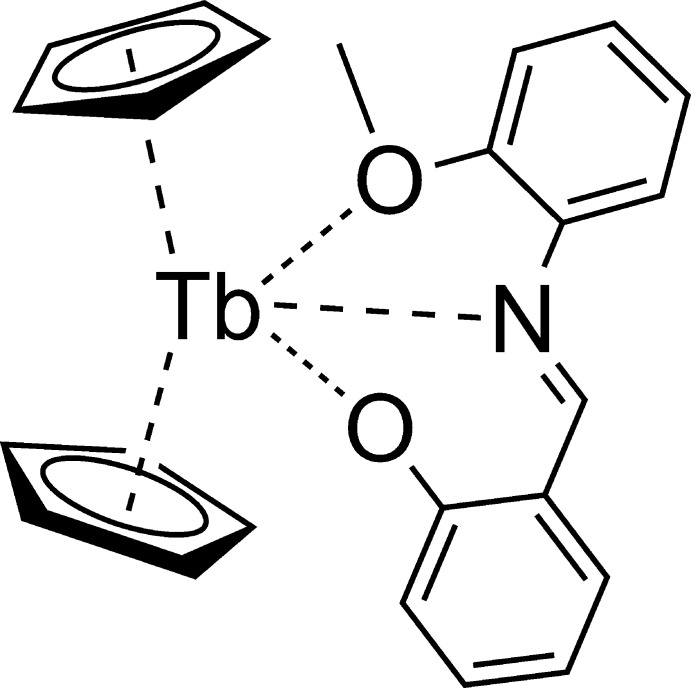


**Figure 1:**
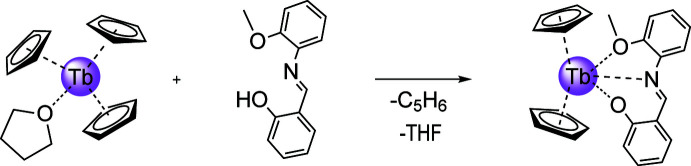
Synthesis of the title compound.

The vigorous inter­est in cyclo­penta­dienyl complexes for the chemistry of rare-earth elements is mainly due to the simplicity of cyclo­penta­dienyl ligand modification by replacing the hydrogen atoms of the five-membered ring with various organic fragments (Harder *et al.*, 2013 ▸; Roitershtein, Puntus *et al.*, 2018 ▸; Hou & Wakatsuki, 2002 ▸). Moreover, in the case of bis­(cyclo­penta­dien­yl) derivatives such as (C_5_H_5_)_2_ *LnX*, the additional anionic ligand *X* ^−^ can be coordinated in a mono-, bi- or, as in the present case, a tridentate mode. Such a combination of ligands provides an extremely broad structural diversity for cyclo­penta­dienyl derivatives (Edelmann & Poremba, 1997 ▸; Goodwin *et al.*, 2018 ▸). This report describes the synthesis and crystal structure of bis­(η^5^-cyclo­penta­dien­yl)(2-{[(2-meth­oxy­phen­yl)imino]­meth­yl}phenolato)terbium, which is a product of the partial protonation of the tris(cyclo­penta­dien­yl)terbium complex with 2-{[(2-meth­oxy­phen­yl)imino]­meth­yl}phenol (Fig. 1 ▸).

## Structural commentary

**Figure 2:**
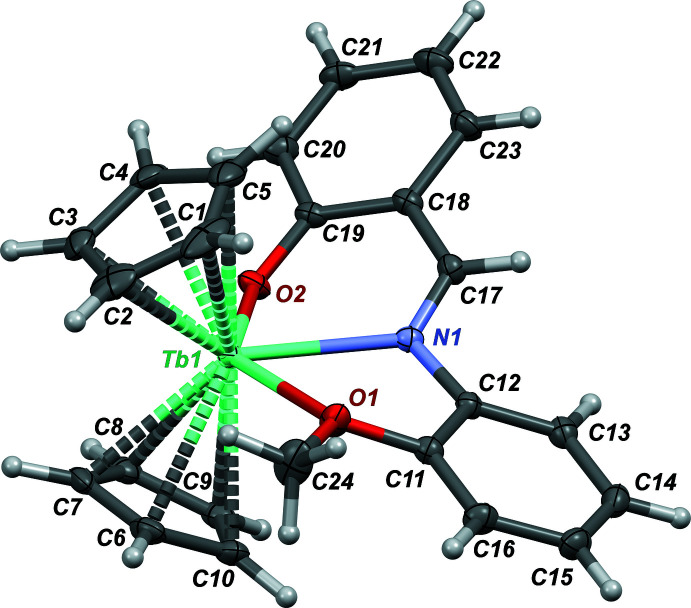
The title compound with displacement ellipsoids drawn at the 50% probability level.

The title compound (Fig. 2 ▸) crystallizes in the ortho­rhom­bic *Pbcn* space group (*Z*′ = 1). Assuming that each cyclo­penta­dienyl ligand donates three electron pairs, the terbium atom may be considered to be *ennea*-coordinated. Both cyclo­penta­dienyl ligands are nearly symmetrically η^5^-coordinated to the Tb^3+^ cation. Thus, the Cp_(centroid)_—Tb distances [2.4207 (11) Å for the C1–C5 Cp ring and 2.4062 (10) Å for the C6–C10 Cp ring] are almost equal to the Cp_(plane)_—Tb distances [2.4196 (11) Å for C1–C5 Cp ring and 2.4054 (10) for C6–C10 Cp ring], and the C_Cp_—Tb bond lengths are similar within each ring (Table 1 ▸). At the same time, the average C_Cp_—Tb distance to the C1–C5 ring is longer by 0.011 Å than to the second Cp ligand. Such a slight asymmetry is caused by the presence of the tridentate asymmetric 2-{[(2-meth­oxy­phen­yl)imino]­meth­yl}phenolate (*L* ^−^) ligand. Atoms of the ligand are situated in two planes formed by the following sets of atoms: O1, C11–C16, N1, C24 (r.m.s. deviation = 0.0167 Å) and O2, C17–C23, N1 (r.m.s. deviation = 0.0333 Å). The dihedral angle between these planes of 44.58 (5)° indicates a perceptible loss of conjugation between two parts of the ligand due to the tridentate κ^3^ *N*,*O*,*O*′-coordination mode. The bond redistribution within the ligand (see table in the supporting information) and the Tb—O and Tb—N bond distances (Table 1 ▸) are in good agreement with the expected predominant resonance form (see scheme) and with a significant localization of the negative charge on the O2 atom.

It should be noted that analogous compounds with the same *L* ^−^ ligand [(C_5_H_5_)_2_ *Ln*(O_2_NC_14_H_12_)] (*Ln* = Sm, Er, Dy, Y) were previously synthesized in low yields (Yousaf *et al.*, 2000 ▸), and the determined crystal structure of the Sm complex is isostructural with that of the title compound.

Non-covalent interactions are negligible in the title compund.

## Database survey

At first glance, it looks quite puzzling that according to the Cambridge Structural Database (CSD Version 5.42, update of September 2021; Groom *et al.*, 2016 ▸), structures of rare-earth metal complexes with the monoanionic phenolate *L* ^−^ ligand and its substituted (*L*′^−^) or protonated (*L*H) derivatives have been poorly studied, whereas the structures of complexes bearing their closest analogs – doubly charged 2-{[(2-oxidophen­yl)imino]­meth­yl}phenolate and its various derivatives – have been studied moderately. This is, likely, due to the higher stability of the latter complexes, which is presumably caused, in short, by a higher degree of the optimization of electrostatic inter­actions (Evans, 1987 ▸). Thus, only 15 complexes bearing *L* ^-^, *L*′^−^, *L*H and L′H ligands have been studied structurally; the corresponding CSD codes are KESHOH (Li & Yuan, 2012 ▸), KINHUN, KINJAV, KINJEZ, KINJID, KINJOJ (Roitershtein, Minashina *et al.*, 2018 ▸), MIQTAH01 (Yousaf *et al.*, 2000 ▸), RAPTUA (Li & Cui, 2017 ▸), RUQQEC (Pikoli *et al.*, 2020 ▸), VUVMUX, VUVNAE (Long *et al.*, 2020 ▸) and the heterometallic Zn/Dy complexes TUQWAG, TUQWEK, TUQWIO, TUQWOU (Shukla *et al.*, 2020 ▸). Careful analysis reveals the structural diversity of the coordination modes for *L* ^−^, *L*′^−^, *L*H and *L*′H ligands in the above-mentioned complexes. Even the sole ligand *L* ^−^ itself can demonstrate different coordination modes in mononuclear rare-earth complexes (Roitershtein, Minashina *et al.*, 2018 ▸). Amazingly, only one structure (MIQTAH01) among the 15 corresponds to the organolanthanide bis­(cyclo­penta­dien­yl) type.

## Synthesis and crystallization

Synthetic operations were carried out in a glovebox under a purified argon atmosphere. THF was distilled from sodium/benzo­phenone ketyl, hexane was distilled from Na/K alloy. Tb(C_5_H_5_)_3_(thf) was obtained according to a literature procedure (Wilkinson & Birmingham, 1954 ▸).

A solution of 2-{[(2-meth­oxy­phen­yl)imino]­meth­yl}phenol (0.230 g, 1.01mmol) in 5 ml of THF was added slowly to a solution of Tb(C_5_H_5_)_3_(thf) (0.426g, 1.0 mmol) in 15 ml of THF. The reaction mixture was stirred for 24 h. The solution was concentrated under vacuum to a volume of *ca* 8–10 ml, and hexane (10 ml) was carefully layered on top of the resulting solution to initiate crystallization. Crystals obtained after several days were dried under dynamic vacuum for 1 h, yielding 0.315 g (0.61 mmol, 61%). The terbium content was determined by direct complexometric titration with the disodium salt of EDTA, using xylenol orange indicator (Vogel, 1966 ▸). Calculated for C_24_H_22_NO_2_Tb: Tb, 30.84%. Found Tb, 30.45%.

Single crystals suitable for X-ray diffraction study were taken from a vial with a crude product before drying under vacuum.

## Refinement

Crystal data, data collection and structure refinement details are summarized in Table 2 ▸. The structure was in general solved by dual methods (*SHELXT*; Sheldrick, 2015*a* ▸). Positions of remaining non-H atoms were found from the difference electron density maps. All non-H atoms were refined anisotropically. The positions of hydrogen atoms were refined with *U*_iso_(H) = 1.5*U*_eq_(C) for methyl group and *U*_iso_(H) = 1.2*U*_eq_(C) for others.

## Supplementary Material

Crystal structure: contains datablock(s) I. DOI: 10.1107/S2056989021013025/yk2162sup1.cif

Structure factors: contains datablock(s) I. DOI: 10.1107/S2056989021013025/yk2162Isup2.hkl

Supporting information file. DOI: 10.1107/S2056989021013025/yk2162Isup3.cdx

CCDC reference: 2127087

Additional supporting information:  crystallographic information; 3D view; checkCIF report

## Figures and Tables

**Table 1:** Selected bond lengths (Å)

Tb1—C1	2.721 (3)	Tb1—C8	2.662 (2)
Tb1—C2	2.678 (3)	Tb1—C9	2.691 (2)
Tb1—C3	2.670 (2)	Tb1—C10	2.717 (2)
Tb1—C4	2.704 (2)	Tb1—O1	2.5468 (15)
Tb1—C5	2.726 (3)	Tb1—O2	2.2034 (16)
Tb1—C6	2.700 (2)	Tb1—N1	2.4748 (18)
Tb1—C7	2.675 (2)		

**Table 2:** Experimental details

Crystal data	
Chemical formula	[Tb(C_5_H_5_)_2_(C_14_H_12_NO_2_)]
*M*_r_	515.34
Crystal system, space group	Orthorhombic, *P* *b* *c* *n*
Temperature (K)	120
*a*, *b*, *c* (Å)	21.6309 (12), 14.4923 (8), 12.6471 (7)
*V* (Å^3^)	3964.6 (4)
*Z*	8
Radiation type	Mo *K*α
μ (mm^−1^)	3.59
Crystal size (mm)	0.32 × 0.21 × 0.19

Data collection	
Diffractometer	Bruker APEXII CCD area detector
Absorption correction	Multi-scan (*SADABS*; Krause *et al.*, 2015 ▸)
*T*_min_, *T*_max_	0.333, 0.569
No. of measured, independent and observed [*I* > 2σ(*I*)] reflections	60268, 7485, 5865
*R*_int_	0.062
(sin θ/λ)_max_ (Å^−1^)	0.766

Refinement	
*R*[*F* ^2^ > 2σ(*F* ^2^)], *wR*(*F* ^2^), *S*	0.025, 0.060, 1.03
No. of reflections	7485
No. of parameters	319
H-atom treatment	Only H-atom coordinates refined
Δρ_max_, Δρ_min_ (e Å^−3^)	1.14, −0.71

Computer programs: *APEX2* and *SAINT* (Bruker, 2016 ▸), *SHELXT* (Sheldrick, 2015*a* ▸), *SHELXL2018* (Sheldrick, 2015*b* ▸), *Mercury* (Macrae *et al.*, 2020 ▸) and *publCIF* (Westrip, 2010 ▸).

## supplementary crystallographic information

### Crystal data

**Table:**

[Tb(C_5_H_5_)_2_(C_14_H_12_NO_2_)]	*D*_x_ = 1.727 Mg m^−^^3^
*M**_r_* = 515.34	Mo *K*α radiation, λ = 0.71073 Å
Orthorhombic, *P**b**c**n*	Cell parameters from 5648 reflections
*a* = 21.6309 (12) Å	θ = 2.2–30.2°
*b* = 14.4923 (8) Å	µ = 3.59 mm^−^^1^
*c* = 12.6471 (7) Å	*T* = 120 K
*V* = 3964.6 (4) Å^3^	Block, yellow
*Z* = 8	0.32 × 0.21 × 0.19 mm
*F*(000) = 2032	

### Data collection

**Table:**

Bruker APEXII CCD area detector diffractometer	7485 independent reflections
Radiation source: sealed X-ray tube	5865 reflections with *I* > 2σ(*I*)
Graphite monochromator	*R*_int_ = 0.062
Detector resolution: 7.31 pixels mm^-1^	θ_max_ = 33.0°, θ_min_ = 1.7°
ω scans	*h* = −33→33
Absorption correction: multi-scan (SADABS; Krause *et al.*, 2015)	*k* = −22→21
*T*_min_ = 0.333, *T*_max_ = 0.569	*l* = −19→19
60268 measured reflections	

### Refinement

**Table:**

Refinement on *F*^2^	Primary atom site location: dual
Least-squares matrix: full	Secondary atom site location: difference Fourier map
*R*[*F*^2^ > 2σ(*F*^2^)] = 0.025	Hydrogen site location: difference Fourier map
*wR*(*F*^2^) = 0.060	Only H-atom coordinates refined
*S* = 1.03	*w* = 1/[σ^2^(*F*_o_^2^) + (0.0212*P*)^2^ + 3.2936*P*] where *P* = (*F*_o_^2^ + 2*F*_c_^2^)/3
7485 reflections	(Δ/σ)_max_ = 0.004
319 parameters	Δρ_max_ = 1.14 e Å^−^^3^
0 restraints	Δρ_min_ = −0.71 e Å^−^^3^

### Special details

**Table:**

Geometry. All esds (except the esd in the dihedral angle between two l.s. planes) are estimated using the full covariance matrix. The cell esds are taken into account individually in the estimation of esds in distances, angles and torsion angles; correlations between esds in cell parameters are only used when they are defined by crystal symmetry. An approximate (isotropic) treatment of cell esds is used for estimating esds involving l.s. planes.
Refinement. Refinement of F^2^ against ALL reflections. The weighted R-factor wR and goodness of fit S are based on F^2^, conventional R-factors R are based on F, with F set to zero for negative F^2^. The threshold expression of F^2^ > 2sigma(F^2^) is used only for calculating R-factors(gt) etc. and is not relevant to the choice of reflections for refinement. R-factors based on F^2^ are statistically about twice as large as those based on F, and R- factors based on ALL data will be even larger.

### Fractional atomic coordinates and isotropic or equivalent isotropic displacement parameters (Å^2^)

**Table:**

	*x*	*y*	*z*	*U*_iso_*/*U*_eq_	
Tb1	0.65689 (2)	0.77625 (2)	0.32099 (2)	0.01273 (3)	
O1	0.61771 (7)	0.82735 (11)	0.50164 (12)	0.0193 (3)	
O2	0.63302 (8)	0.67000 (11)	0.20325 (12)	0.0190 (3)	
N1	0.56840 (8)	0.68915 (12)	0.39465 (14)	0.0144 (3)	
C1	0.74352 (13)	0.7329 (2)	0.4689 (2)	0.0378 (7)	
H1	0.7343 (16)	0.741 (2)	0.539 (3)	0.045*	
C2	0.77065 (12)	0.7984 (2)	0.4006 (2)	0.0328 (6)	
H2	0.7832 (15)	0.859 (2)	0.415 (3)	0.039*	
C3	0.77899 (11)	0.75538 (19)	0.3010 (2)	0.0259 (5)	
H3	0.7968 (14)	0.786 (2)	0.242 (3)	0.031*	
C4	0.75698 (12)	0.66501 (19)	0.3091 (2)	0.0263 (5)	
H4	0.7546 (14)	0.620 (2)	0.256 (2)	0.032*	
C5	0.73448 (13)	0.6512 (2)	0.4122 (2)	0.0337 (6)	
H5	0.7166 (15)	0.594 (2)	0.432 (3)	0.040*	
C6	0.65210 (11)	0.96160 (15)	0.3007 (2)	0.0225 (5)	
H6	0.6687 (13)	0.996 (2)	0.354 (2)	0.027*	
C7	0.68593 (11)	0.92900 (15)	0.2132 (2)	0.0223 (5)	
H7	0.7266 (14)	0.941 (2)	0.203 (2)	0.027*	
C8	0.64535 (11)	0.87859 (16)	0.1473 (2)	0.0209 (4)	
H8	0.6550 (12)	0.851 (2)	0.083 (2)	0.025*	
C9	0.58601 (11)	0.88127 (15)	0.19414 (19)	0.0201 (4)	
H9	0.5500 (14)	0.850 (2)	0.168 (2)	0.024*	
C10	0.59022 (11)	0.93258 (15)	0.2884 (2)	0.0208 (4)	
H10	0.5580 (14)	0.943 (2)	0.334 (2)	0.025*	
C11	0.55636 (10)	0.80986 (14)	0.52484 (17)	0.0157 (4)	
C12	0.53032 (10)	0.73486 (13)	0.47039 (16)	0.0141 (4)	
C13	0.46845 (10)	0.71343 (15)	0.48786 (17)	0.0180 (4)	
H13	0.4491 (12)	0.6662 (19)	0.448 (2)	0.022*	
C14	0.43354 (11)	0.76366 (17)	0.55984 (19)	0.0224 (5)	
H14	0.3905 (14)	0.750 (2)	0.569 (2)	0.027*	
C15	0.46002 (11)	0.83688 (17)	0.61333 (19)	0.0227 (5)	
H15	0.4355 (14)	0.875 (2)	0.657 (2)	0.027*	
C16	0.52167 (11)	0.86056 (16)	0.59584 (18)	0.0209 (4)	
H16	0.5394 (13)	0.907 (2)	0.632 (2)	0.025*	
C17	0.55750 (10)	0.60229 (15)	0.37651 (17)	0.0165 (4)	
H17	0.5284 (12)	0.5710 (19)	0.420 (2)	0.020*	
C18	0.58451 (10)	0.54572 (14)	0.29562 (16)	0.0158 (4)	
C19	0.61933 (10)	0.58261 (14)	0.20989 (17)	0.0157 (4)	
C20	0.63711 (12)	0.52071 (16)	0.12895 (18)	0.0204 (4)	
H20	0.6584 (12)	0.547 (2)	0.071 (2)	0.025*	
C21	0.62280 (12)	0.42804 (16)	0.1339 (2)	0.0221 (5)	
H21	0.6372 (14)	0.391 (2)	0.081 (2)	0.027*	
C22	0.58962 (12)	0.39198 (16)	0.2186 (2)	0.0235 (5)	
H22	0.5803 (13)	0.332 (2)	0.222 (2)	0.028*	
C23	0.57048 (12)	0.45061 (15)	0.29754 (19)	0.0213 (4)	
H23	0.5481 (13)	0.431 (2)	0.356 (2)	0.026*	
C24	0.64613 (13)	0.9021 (2)	0.5605 (2)	0.0310 (6)	
H24A	0.6895 (17)	0.903 (2)	0.538 (3)	0.046*	
H24B	0.6416 (16)	0.886 (3)	0.632 (3)	0.046*	
H24C	0.6246 (16)	0.961 (3)	0.547 (3)	0.046*	

### Atomic displacement parameters (Å^2^)

**Table:**

	*U*^11^	*U*^22^	*U*^33^	*U*^12^	*U*^13^	*U*^23^
Tb1	0.01162 (5)	0.01062 (4)	0.01596 (5)	0.00074 (3)	−0.00014 (3)	0.00054 (3)
O1	0.0168 (7)	0.0194 (7)	0.0216 (8)	−0.0033 (6)	0.0009 (6)	−0.0079 (6)
O2	0.0274 (8)	0.0121 (6)	0.0176 (7)	−0.0002 (6)	0.0015 (6)	0.0001 (6)
N1	0.0164 (8)	0.0135 (8)	0.0135 (8)	0.0009 (6)	−0.0009 (6)	−0.0010 (6)
C1	0.0269 (13)	0.065 (2)	0.0213 (12)	0.0232 (14)	−0.0049 (10)	0.0018 (13)
C2	0.0179 (11)	0.0381 (15)	0.0423 (16)	0.0055 (10)	−0.0116 (11)	−0.0095 (12)
C3	0.0139 (10)	0.0319 (12)	0.0319 (14)	0.0049 (9)	0.0009 (9)	0.0066 (10)
C4	0.0198 (11)	0.0269 (12)	0.0320 (13)	0.0116 (9)	0.0036 (10)	0.0012 (10)
C5	0.0260 (13)	0.0354 (15)	0.0397 (15)	0.0159 (11)	0.0072 (11)	0.0163 (12)
C6	0.0237 (11)	0.0112 (9)	0.0325 (13)	−0.0014 (8)	−0.0047 (9)	−0.0005 (8)
C7	0.0174 (10)	0.0147 (9)	0.0346 (13)	−0.0013 (8)	0.0004 (9)	0.0095 (9)
C8	0.0255 (12)	0.0155 (10)	0.0216 (10)	0.0045 (8)	0.0013 (9)	0.0045 (8)
C9	0.0192 (10)	0.0136 (9)	0.0273 (12)	0.0011 (8)	−0.0046 (9)	0.0015 (8)
C10	0.0203 (11)	0.0142 (9)	0.0280 (11)	0.0036 (8)	0.0018 (9)	0.0000 (8)
C11	0.0167 (9)	0.0151 (9)	0.0153 (9)	0.0001 (7)	−0.0011 (7)	−0.0015 (7)
C12	0.0162 (9)	0.0141 (9)	0.0119 (8)	0.0002 (7)	0.0006 (7)	0.0016 (7)
C13	0.0161 (9)	0.0200 (10)	0.0179 (10)	−0.0028 (8)	−0.0008 (8)	0.0028 (8)
C14	0.0176 (10)	0.0267 (12)	0.0229 (11)	0.0015 (9)	0.0031 (8)	0.0054 (9)
C15	0.0248 (12)	0.0221 (11)	0.0213 (11)	0.0041 (9)	0.0067 (9)	−0.0007 (9)
C16	0.0258 (12)	0.0193 (10)	0.0175 (10)	0.0017 (9)	0.0010 (9)	−0.0039 (8)
C17	0.0182 (10)	0.0154 (9)	0.0160 (9)	−0.0015 (8)	0.0002 (8)	−0.0006 (7)
C18	0.0189 (10)	0.0131 (9)	0.0155 (9)	0.0000 (7)	−0.0015 (7)	−0.0006 (7)
C19	0.0191 (10)	0.0127 (9)	0.0153 (9)	0.0014 (7)	−0.0027 (8)	−0.0003 (7)
C20	0.0265 (11)	0.0183 (10)	0.0165 (10)	0.0033 (9)	0.0004 (9)	−0.0024 (8)
C21	0.0260 (12)	0.0173 (10)	0.0230 (11)	0.0048 (9)	−0.0029 (9)	−0.0071 (9)
C22	0.0285 (12)	0.0136 (10)	0.0286 (12)	−0.0002 (9)	−0.0041 (10)	−0.0042 (9)
C23	0.0265 (12)	0.0136 (9)	0.0237 (11)	−0.0020 (8)	0.0012 (9)	−0.0005 (8)
C24	0.0270 (13)	0.0335 (14)	0.0324 (14)	−0.0105 (11)	0.0005 (11)	−0.0180 (11)

### Geometric parameters (Å, º)

**Table:**

Tb1—C1	2.721 (3)	C7—H7	0.91 (3)
Tb1—C2	2.678 (3)	C8—C9	1.414 (3)
Tb1—C3	2.670 (2)	C8—H8	0.93 (3)
Tb1—C4	2.704 (2)	C9—C10	1.408 (3)
Tb1—C5	2.726 (3)	C9—H9	0.96 (3)
Tb1—C6	2.700 (2)	C10—H10	0.91 (3)
Tb1—C7	2.675 (2)	C11—C16	1.382 (3)
Tb1—C8	2.662 (2)	C11—C12	1.405 (3)
Tb1—C9	2.691 (2)	C12—C13	1.392 (3)
Tb1—C10	2.717 (2)	C13—C14	1.389 (3)
Tb1—O1	2.5468 (15)	C13—H13	0.95 (3)
Tb1—O2	2.2034 (16)	C14—C15	1.383 (4)
Tb1—N1	2.4748 (18)	C14—H14	0.96 (3)
O1—C11	1.383 (3)	C15—C16	1.395 (3)
O1—C24	1.451 (3)	C15—H15	0.95 (3)
O2—C19	1.303 (3)	C16—H16	0.90 (3)
N1—C17	1.301 (3)	C17—C18	1.435 (3)
N1—C12	1.427 (3)	C17—H17	0.95 (3)
C1—C5	1.398 (5)	C18—C23	1.412 (3)
C1—C2	1.411 (5)	C18—C19	1.424 (3)
C1—H1	0.91 (4)	C19—C20	1.414 (3)
C2—C3	1.417 (4)	C20—C21	1.380 (3)
C2—H2	0.94 (3)	C20—H20	0.95 (3)
C3—C4	1.397 (4)	C21—C22	1.392 (4)
C3—H3	0.94 (3)	C21—H21	0.91 (3)
C4—C5	1.406 (4)	C22—C23	1.375 (3)
C4—H4	0.93 (3)	C22—H22	0.90 (3)
C5—H5	0.95 (3)	C23—H23	0.92 (3)
C6—C7	1.408 (4)	C24—H24A	0.98 (4)
C6—C10	1.412 (3)	C24—H24B	0.94 (4)
C6—H6	0.92 (3)	C24—H24C	0.98 (4)
C7—C8	1.413 (4)		
			
O2—Tb1—N1	73.54 (6)	C3—C2—H2	123 (2)
O2—Tb1—O1	137.01 (6)	Tb1—C2—H2	117 (2)
N1—Tb1—O1	63.47 (5)	C4—C3—C2	107.7 (2)
O2—Tb1—C8	79.04 (7)	C4—C3—Tb1	76.25 (14)
N1—Tb1—C8	121.49 (7)	C2—C3—Tb1	74.94 (14)
O1—Tb1—C8	123.16 (6)	C4—C3—H3	129.4 (19)
O2—Tb1—C3	95.09 (8)	C2—C3—H3	122.9 (19)
N1—Tb1—C3	137.96 (7)	Tb1—C3—H3	115.1 (19)
O1—Tb1—C3	116.54 (7)	C3—C4—C5	108.6 (3)
C8—Tb1—C3	94.46 (8)	C3—C4—Tb1	73.62 (14)
O2—Tb1—C7	106.80 (7)	C5—C4—Tb1	75.87 (14)
N1—Tb1—C7	142.72 (7)	C3—C4—H4	128.1 (19)
O1—Tb1—C7	107.14 (7)	C5—C4—H4	123.2 (19)
C8—Tb1—C7	30.72 (8)	Tb1—C4—H4	114.2 (19)
C3—Tb1—C7	79.24 (8)	C1—C5—C4	107.9 (3)
O2—Tb1—C2	123.58 (8)	C1—C5—Tb1	74.94 (15)
N1—Tb1—C2	129.07 (8)	C4—C5—Tb1	74.11 (14)
O1—Tb1—C2	86.20 (8)	C1—C5—H5	131 (2)
C8—Tb1—C2	109.23 (9)	C4—C5—H5	121 (2)
C3—Tb1—C2	30.71 (9)	Tb1—C5—H5	116 (2)
C7—Tb1—C2	82.91 (9)	C7—C6—C10	107.9 (2)
O2—Tb1—C9	81.89 (6)	C7—C6—Tb1	73.80 (13)
N1—Tb1—C9	94.15 (6)	C10—C6—Tb1	75.54 (13)
O1—Tb1—C9	100.42 (6)	C7—C6—H6	124.0 (18)
C8—Tb1—C9	30.63 (7)	C10—C6—H6	128.1 (18)
C3—Tb1—C9	124.84 (8)	Tb1—C6—H6	117.5 (19)
C7—Tb1—C9	50.34 (7)	C6—C7—C8	108.3 (2)
C2—Tb1—C9	132.83 (9)	C6—C7—Tb1	75.84 (13)
O2—Tb1—C6	128.45 (7)	C8—C7—Tb1	74.15 (13)
N1—Tb1—C6	120.91 (7)	C6—C7—H7	123.9 (18)
O1—Tb1—C6	77.49 (7)	C8—C7—H7	127.8 (18)
C8—Tb1—C6	50.48 (8)	Tb1—C7—H7	117.4 (19)
C3—Tb1—C6	98.14 (8)	C7—C8—C9	107.6 (2)
C7—Tb1—C6	30.36 (8)	C7—C8—Tb1	75.14 (13)
C2—Tb1—C6	87.23 (8)	C9—C8—Tb1	75.82 (13)
C9—Tb1—C6	50.12 (7)	C7—C8—H8	126.8 (17)
O2—Tb1—C4	74.52 (7)	C9—C8—H8	125.6 (17)
N1—Tb1—C4	109.64 (7)	Tb1—C8—H8	117.0 (18)
O1—Tb1—C4	119.34 (7)	C10—C9—C8	108.1 (2)
C8—Tb1—C4	111.18 (8)	C10—C9—Tb1	75.93 (13)
C3—Tb1—C4	30.13 (8)	C8—C9—Tb1	73.55 (13)
C7—Tb1—C4	106.08 (8)	C10—C9—H9	126.6 (17)
C2—Tb1—C4	49.96 (9)	C8—C9—H9	125.3 (17)
C9—Tb1—C4	139.49 (8)	Tb1—C9—H9	113.8 (17)
C6—Tb1—C4	128.21 (8)	C9—C10—C6	108.1 (2)
O2—Tb1—C10	110.85 (7)	C9—C10—Tb1	73.89 (13)
N1—Tb1—C10	94.13 (7)	C6—C10—Tb1	74.25 (13)
O1—Tb1—C10	73.55 (6)	C9—C10—H10	124.7 (18)
C8—Tb1—C10	50.25 (7)	C6—C10—H10	127.2 (18)
C3—Tb1—C10	127.23 (8)	Tb1—C10—H10	116.7 (18)
C7—Tb1—C10	50.00 (7)	C16—C11—O1	124.17 (19)
C2—Tb1—C10	116.40 (8)	C16—C11—C12	120.8 (2)
C9—Tb1—C10	30.19 (7)	O1—C11—C12	115.01 (18)
C6—Tb1—C10	30.20 (7)	C13—C12—C11	118.72 (19)
C4—Tb1—C10	156.04 (8)	C13—C12—N1	123.94 (19)
O2—Tb1—C1	117.67 (8)	C11—C12—N1	117.20 (19)
N1—Tb1—C1	98.98 (8)	C14—C13—C12	120.7 (2)
O1—Tb1—C1	71.32 (7)	C14—C13—H13	119.3 (16)
C8—Tb1—C1	139.49 (9)	C12—C13—H13	120.0 (16)
C3—Tb1—C1	50.03 (9)	C15—C14—C13	119.8 (2)
C7—Tb1—C1	112.31 (9)	C15—C14—H14	120.3 (18)
C2—Tb1—C1	30.29 (10)	C13—C14—H14	119.8 (18)
C9—Tb1—C1	158.87 (9)	C14—C15—C16	120.5 (2)
C6—Tb1—C1	108.76 (9)	C14—C15—H15	120.2 (19)
C4—Tb1—C1	49.39 (9)	C16—C15—H15	119.0 (19)
C10—Tb1—C1	131.47 (9)	C11—C16—C15	119.4 (2)
O2—Tb1—C5	88.02 (8)	C11—C16—H16	119.8 (19)
N1—Tb1—C5	88.73 (7)	C15—C16—H16	120.8 (18)
O1—Tb1—C5	91.07 (7)	N1—C17—C18	127.2 (2)
C8—Tb1—C5	141.00 (8)	N1—C17—H17	118.6 (16)
C3—Tb1—C5	49.89 (8)	C18—C17—H17	114.2 (16)
C7—Tb1—C5	128.41 (8)	C23—C18—C19	119.6 (2)
C2—Tb1—C5	49.83 (10)	C23—C18—C17	117.3 (2)
C9—Tb1—C5	168.24 (8)	C19—C18—C17	122.92 (19)
C6—Tb1—C5	136.49 (9)	O2—C19—C20	120.5 (2)
C4—Tb1—C5	30.02 (8)	O2—C19—C18	122.27 (19)
C10—Tb1—C5	160.96 (9)	C20—C19—C18	117.18 (19)
C1—Tb1—C5	29.74 (10)	C21—C20—C19	121.6 (2)
C11—O1—C24	115.77 (18)	C21—C20—H20	122.1 (19)
C11—O1—Tb1	117.16 (12)	C19—C20—H20	116.3 (19)
C24—O1—Tb1	122.42 (15)	C20—C21—C22	121.1 (2)
C19—O2—Tb1	133.53 (14)	C20—C21—H21	117.7 (19)
C17—N1—C12	117.59 (18)	C22—C21—H21	121.1 (19)
C17—N1—Tb1	124.56 (15)	C23—C22—C21	118.8 (2)
C12—N1—Tb1	117.54 (12)	C23—C22—H22	120.3 (19)
C5—C1—C2	108.3 (3)	C21—C22—H22	120.9 (19)
C5—C1—Tb1	75.32 (16)	C22—C23—C18	121.8 (2)
C2—C1—Tb1	73.17 (15)	C22—C23—H23	123.1 (18)
C5—C1—H1	125 (2)	C18—C23—H23	115.1 (18)
C2—C1—H1	127 (2)	O1—C24—H24A	105 (2)
Tb1—C1—H1	119 (2)	O1—C24—H24B	105 (2)
C1—C2—C3	107.5 (3)	H24A—C24—H24B	113 (3)
C1—C2—Tb1	76.54 (16)	O1—C24—H24C	111 (2)
C3—C2—Tb1	74.34 (14)	H24A—C24—H24C	113 (3)
C1—C2—H2	129 (2)	H24B—C24—H24C	110 (3)
			
C5—C1—C2—C3	−0.8 (3)	Tb1—O1—C11—C12	26.1 (2)
Tb1—C1—C2—C3	−68.51 (18)	C16—C11—C12—C13	1.1 (3)
C5—C1—C2—Tb1	67.73 (19)	O1—C11—C12—C13	−178.98 (18)
C1—C2—C3—C4	0.2 (3)	C16—C11—C12—N1	176.94 (19)
Tb1—C2—C3—C4	−69.85 (18)	O1—C11—C12—N1	−3.2 (3)
C1—C2—C3—Tb1	70.01 (18)	C17—N1—C12—C13	−32.4 (3)
C2—C3—C4—C5	0.5 (3)	Tb1—N1—C12—C13	153.66 (16)
Tb1—C3—C4—C5	−68.45 (18)	C17—N1—C12—C11	152.0 (2)
C2—C3—C4—Tb1	68.95 (17)	Tb1—N1—C12—C11	−21.9 (2)
C2—C1—C5—C4	1.1 (3)	C11—C12—C13—C14	−1.7 (3)
Tb1—C1—C5—C4	67.38 (18)	N1—C12—C13—C14	−177.2 (2)
C2—C1—C5—Tb1	−66.30 (19)	C12—C13—C14—C15	1.3 (3)
C3—C4—C5—C1	−1.0 (3)	C13—C14—C15—C16	−0.2 (4)
Tb1—C4—C5—C1	−67.94 (19)	O1—C11—C16—C15	180.0 (2)
C3—C4—C5—Tb1	66.96 (18)	C12—C11—C16—C15	−0.1 (3)
C10—C6—C7—C8	−0.8 (3)	C14—C15—C16—C11	−0.3 (4)
Tb1—C6—C7—C8	67.66 (16)	C12—N1—C17—C18	171.9 (2)
C10—C6—C7—Tb1	−68.48 (16)	Tb1—N1—C17—C18	−14.6 (3)
C6—C7—C8—C9	0.7 (3)	N1—C17—C18—C23	173.7 (2)
Tb1—C7—C8—C9	69.50 (16)	N1—C17—C18—C19	−12.3 (4)
C6—C7—C8—Tb1	−68.79 (16)	Tb1—O2—C19—C20	−146.18 (18)
C7—C8—C9—C10	−0.3 (3)	Tb1—O2—C19—C18	36.0 (3)
Tb1—C8—C9—C10	68.71 (16)	C23—C18—C19—O2	179.2 (2)
C7—C8—C9—Tb1	−69.03 (16)	C17—C18—C19—O2	5.2 (3)
C8—C9—C10—C6	−0.2 (3)	C23—C18—C19—C20	1.3 (3)
Tb1—C9—C10—C6	66.92 (16)	C17—C18—C19—C20	−172.6 (2)
C8—C9—C10—Tb1	−67.12 (16)	O2—C19—C20—C21	−179.6 (2)
C7—C6—C10—C9	0.6 (3)	C18—C19—C20—C21	−1.6 (3)
Tb1—C6—C10—C9	−66.68 (16)	C19—C20—C21—C22	0.7 (4)
C7—C6—C10—Tb1	67.31 (16)	C20—C21—C22—C23	0.7 (4)
C24—O1—C11—C16	2.4 (3)	C21—C22—C23—C18	−1.1 (4)
Tb1—O1—C11—C16	−154.05 (18)	C19—C18—C23—C22	0.1 (4)
C24—O1—C11—C12	−177.5 (2)	C17—C18—C23—C22	174.3 (2)
